# Supplementary material for: A Long‐Term Ecological Research Data Set From the Marine Genetic Monitoring Program ARMS‐MBON 2018–2020
Source: Mol Ecol Resour. 2025 Jan 31;25(4):e14073. doi: 10.1111/1755-0998.14073 (PMC11969632; doi:10.1111/1755-0998.14073)
Supplement: Supplementary file 5 — Data S1. [file MEN-25-e14073-s004.pdf]

Supplementary Material to

A long-term ecological research data set from the genetic monitoring program ARMS-MBON 2018-2020

Nauras Daraghmeh[1,2], Katrina Exter[3], Justine Pagnier[2,4], Piotr Bałazy[5], Ibon Cancio[6], Giorgos Chatzigeorgiou[7], Eva Chatzinikolaou[7], Maciej Chełchowski[5], Nathan Alexis Mitchell Chrismas[8], Thierry Comtet[9], Thanos Dailianis[7], Klaas Deneudt[3], Oihane Diaz de Cerio[6], Markos Digenis[7,10], Vasilis Gerovasileiou[7,10], José González[11], Laura Kauppi[12], Jon Bent Kristoffersen[7], Piotr Kukliński[5], Rafał Lasota[13], Liraz Levy[14], Magdalena Małachowicz[5], Borut Mavrič[15], Jonas Mortelmans[3], Estefania Paredes[11], Anita Poćwierz-Kotus[5], Henning Reiss[16], Ioulia Santi[7,17], Georgia Sarafidou[7], Grigorios Skouradakis[7], Jostein Solbakken[12], Peter A.U. Staehr[18], Javier Tajadura[6], Jakob Thyrring[18], Jesus S. Troncoso[11], Emmanouela Vernadou[7], Frederique Viard[19], Haris Zafeiropoulos[7,20], Małgorzata Zbawicka[5], Christina Pavloudi[7,17*], Matthias Obst[1,2*+]

Supplementary Texts S1 to S2

Text S1: Bioinformatics processing of raw sequence data

Sequence data were processed with the Pipeline for Environmental DNA Metabarcoding Analysis, PEMA v.2.1.4 (Zafeiropoulos et al., 2020). PEMA currently supports the analysis of five marker genes (12S, 16S, 18S rRNA, COI, and ITS), and may also be used for other genes of interest if a taxonomic reference database is provided. PEMA consists of four main steps: (i) sequence pre-processing, (ii) OTU clustering or ASV inference, (iii) taxonomic assignment, and (iv) optionally the performance of biodiversity analysis based on the taxonomic inventory retrieved. It has been shown that parameter settings can lead to rather different outcomes (Brandt et al., 2021; Zafeiropoulos et al., 2020). Therefore, for comparison reasons, a fixed set of parameters was used for each marker gene and sequence data were processed separately for each sequencing run. All parameter files with their specific settings used as input for PEMA runs are available on the ARMS-MBON GitHub repository (see Supplementary Table S1 for respective link).

Quality of sequence reads was first assessed using FASTQC v0.11.8 (Andrews, 2010). Subsequently, Trimmomatic v0.38 (Bolger et al., 2014) was used to: (i) trim reads applying targetLength = 150 for 18S and ITS and targetLength = 200 for COI to specify the read length which is likely to allow the location of the read within the target sequence to be determined, and with strictness ranging between 0.6 and 0.8 to specify the balance between preserving as much read length as possible vs. removal of incorrect bases; (ii) trim adapter and primer sequences applying seedMismatches = 2, palindromeClipThreshold = 30, and simpleClipThreshold = 15, (iii) trim low quality bases from the beginning (base calls with

quality scores of below 10) and end (base calls with quality scores of below 15) of reads; and (iv) remove reads showing a length of less than 50 (i.e., for 18S) and 100 (i.e., for COI and ITS) bases after the preceding trimming steps. Error correction of reads was performed using BayesHammer (Nikolenko et al., 2013) as part of SPAdes v3.13.0 (Bankevich et al., 2012). Merging of paired reads was done applying the "simple_bayesian" algorithm in PANDAseq v2.11 (Masella et al., 2012). Here, the minimum read length was set to 50 for 18S and 150 for COI and ITS, the minimum overlap to 5 (18S), 10 (ITS) and 20 (COI) nucleotides, and the required quality threshold to 0.6 for all marker genes. Reads with ambiguous base calls (i.e., N calls) remaining were removed. Subsequently, the "obiuniq" program of OBITools v1.2.12 (Boyer et al., 2016) was used to dereplicate reads.

For the 18S marker gene, sequences were clustered into operational taxonomic units (OTUs) using the VSEARCH v2.9.1 algorithm (Rognes et al., 2016) with a threshold of 0.97, while for ITS and COI, clustering was performed with Swarm v2 (Mahe et al., 2015), applying a threshold of d = 10 to infer amplicon sequence variants (ASVs). Note that PEMA initially defined the result of Swarm processing as inferred ASVs, i.e., sequences which differ by one or more nucleotides, which is now corrected to swarm-clusters (Hakimzadeh et al., 2023). We here use PEMA's terminology for consistency reasons. Taxonomy was assigned to 18S OTUs and ITS ASVs with the CREST LCAClassifier v3.0 (Lanzèn et al., 2012), using the PR2 v.4.13.0 (Guillou et al., 2013) and Unite v7.2 (Nilsson et al., 2018) databases, respectively. Default settings were: i) minimum bit-score = 155; ii) LCA bitscore range = 2%; and iii) similarity cut-offs of 99%, 97%, 95%, 90%, 85% and 80% for the species, genus, family, order, class and phylum ranks, respectively. For COI sequences, taxonomic annotation was performed using the RDP classifier (Wang et al., 2007) with the MIDORI database v2.0 (Machida et al., 2017), and confidence values for each rank assignment were recorded, but no threshold was applied. Singletons (sequences with a total read abundance of one), OTUs/ASVs unclassified at domain level, and potential contaminant sequences (OTUs/ASVs that were more abundant in the negative control samples compared to actual samples) were removed. For OTUs/ASVs that were present in negative control samples in lower abundances than in actual samples, their corresponding read number in the negative controls was subtracted from their read number in actual samples. All bioinformatics analyses were supported by the High Performance Computing system of the Institute of Marine Biology, Biotechnology and Aquaculture of HCMR (Crete, Greece) (Zafeiropoulos et al., 2021).

Text S2: Exploration of sequencing data

The data from individual PEMA runs we provide on GitHub were merged for each marker gene and further curated to obtain a data set for visualisations and ecological assessments. As no confidence threshold was applied within PEMA for taxonomic assignments of COI ASVs (note that this is therefore also the case for the EurOBIS submission and users are urged to apply their own self-chosen cut-off), we excluded all rank assignments with a confidence value of below 0.8 for this marker gene. We further removed the sediment and plankton samples to solely assess the ARMS mobile and sessile data. Subsequently, we removed replicates of certain samples to reduce diversity inflation of the data set: i) where samples were re-sequenced and both versions of the respective sample pair remained in the data set, rarefaction curves, ASV/OTU count tables and taxonomic profiles of those samples were assessed and the version with higher diversity and/or better taxonomic resolution was kept; ii) for cases where samples remaining in the data set were preserved as duplicates in both EtOH and DMSO, only the DMSO sample was kept, as this is now the standard preservative used within ARMS-MBON; and iii) for the biological sample from the Roscoff (France) observatory which was processed as a technical duplicate (i.e., two replicates with the same preservative), rarefaction curves were generated and only the sample with the higher sequence richness and read count was kept.

Sequences with one of the following classifications were discarded as potential contaminants: *Homo sapiens*, *Canis lupus*, *Bos* spp. and *Zea mays*. Sequences classified as Insecta were removed if their lowest rank assignment was not listed in the World Register of Marine Species (WoRMS; Ahyong et al., 2024) as a marine and/or brackish taxon. See Supplementary File 4 for taxonomy and read abundances of removed sequences. One ASV in the ITS data identified as *Petrophila incerta* was incorrectly classified as the insect genus of the same name in the Unite database. We corrected its taxonomy to the *Petrophila* genus of the fungal Extremaceae family (Ascomycota), as this is a fungal species.

We determined the number and relative read abundance of unique phyla recovered through the application of the three marker genes, as well as the number of i) ASVs/OTUs classified with a Linnaean species name, ii) unique species identified, iii) species identified within each phylum, and iv) species shared between the data sets of the three marker genes. As the deposited taxonomy of different taxa does not necessarily follow the same Linnaean classification ranks within and across reference databases, some of the assigned taxonomies did not have a correct phylum level classification. For these cases, we manually determined the actual phylum classification of each infra-/subphylum, class, order, etc. present in our data set through a web-based search. We retrieved the correct phylum name from WoRMS; where this information

could not be found in WoRMS, we relied on further scientific literature. In terms of alpha diversity, we assessed the observed ASV/OTU richness and the number of identified species (at the classification confidence threshold applied here) across observatories, as well as frequency distributions of these two parameters (i.e., re-occurrence of ASVs/OTUs and species identified across observatories).

We also assessed the influence of sampling effort on diversity variables. Here, we computed Spearman's correlation of sequencing depth (i.e., read number) and ARMS deployment duration (measured in days) versus ASV/OTU richness and the number of species identified in each sample. Furthermore, we computed Spearman's correlation of the number of ARMS units deployed and the number of samples included in the analysis post-curation versus ASV/OTU richness and the number of species identified at each of the 15 observatories. Where the correlation was statistically significant (i.e., $p < 0.05$) and moderate to strong (i.e., Spearman's $\rho > 0.4$), we performed analysis of simple linear regression to model the relationship between sampling effort predictor variables (i.e., sequencing depth measured as read numbers for sample-wise data and the number of ARMS units deployed and number of samples analysed for observatory-wise data) and dependent variables (i.e., ASV/OTU richness and number of species identified). As no significant monotonic association was revealed between deployment duration and diversity variables, we applied generalised additive mixed modelling (GAMM; with the fixed effect *Deployment_Days* as smoother and the factor *Observatory* as random effect, 15 levels) for this specific case to test if an alternative significant relation was present.

In order to test the application potential of the derived species observation data, we performed a scan against reference checklists for ecological key species. For this, we pooled COI and 18S data of species occurrences with at least two sequence reads and derived a list of species identified from each ARMS unit for each observatory. This list was then scanned against the following databases: i) AZTI's Marine Biotic Index (AMBI; Borja et al., 2000, 2019) for species very sensitive to disturbance; ii) the World Register of Introduced Marine Species (WRiMS; Costello et al., 2021, 2024) for species with alien status at the place of observation; and iii) the Red Lists of the International Union for Conservation of Nature (IUCN) and Baltic Marine Environment Protection Commission (Helsinki Commission, HELCOM) for species registered as Near Threatened, Vulnerable, Endangered or Critically Endangered. To this end, we used the web services provided by WoRMS. The AMBI and IUCN/HELCOM information were obtained using the WoRMS REST services (https://www.marinespecies.org/rest/; more specifically the call AphiaAttributesByAphiaID), while the WRiMS checks can be replicated using the Jupyter notebook on https://www.github.com/vliz-be-opsci/lw-iji-invasive-checker.

We confirmed occurrences of red-listed species by scanning against known distribution in WoRMS and removed the *Pinna nobilis* occurrence from the Plymouth (UK) observatory, as this species is only known from the Mediterranean Sea and showed a low prevalence of only five reads in the respective sample.

Samples were tested for differences in alpha diversity among locations with varying degrees of anthropogenic influence (i.e., industrial, semi-industrial, low human influence (LHI), and protected; see Supplementary File S1 for influence category of each ARMS unit). After evaluating the deployment locations by consulting each network member, we identified these four categories appropriately describing the anthropogenic influence at each deployment site. Network members then classified each site according to the best-fitting category. For statistical comparison, samples with less than 5,000 reads were removed and the remaining samples rarefied to an equal sequencing depth of 5,000 reads without replacement to reduce diversity bias due to differences in sequencing depth. Given the relatively low number of remaining samples classified as "industrial" (i.e., n = 4 for COI, and n = 6 for 18S), these samples were grouped into one category ("industrial/semi-industrial") with samples classified as "semi-industrial". Mean and standard deviation (SD) of the two alpha diversity measures were calculated for samples of each influence type (i.e., three levels: industrial/semi-industrial, low human influence, and protected) and values were rounded to the nearest whole number. Data was subsequently checked for normality using the Shapiro-Wilk test. If data were normally distributed or log(1+x)-transformation resulted in normality (i.e., for $p > 0.05$), unidirectional analysis of variance (ANOVA) was applied to test for statistically significant differences between habitats. In case of significant differences (i.e., for $p < 0.05$), post-hoc Tukey's test was performed for pairwise comparisons. Where data was not normally distributed and transformation did not achieve normality, non-parametric Kruskal-Wallis rank sum test was applied (no statistical differences were revealed with this test, hence, no post-hoc test was performed).

As described above, all code used for exploratory analysis can be found at the dedicated GitHub repository (see Supplementary Table S1 for link). Analyses and data visualisation were performed in R v4.1.0 (R Core Team, 2021) via RStudio v2022.07.1 (RStudio Team, 2022) using packages of the tidyverse v1.3.1 collection (Wickham et al., 2019) and the packages Biostrings v2.60.2 (Pagès et al., 2020), phyloseq v1.36.0 (McMurdie & Holmes, 2013), vegan v2.6.2 (Oksanen et al., 2023), gamm4 v0.2.6 (Wood & Scheipl, 2020), ggpubr v0.4.0 (Kassambara, 2020), grafify v4.0 (Shenoy, 2021), plyr v1.8.7 (Wickham, 2011), scales v1.3.0 (Wickham et al., 2023), egg v0.4.5 (Auguie, 2019), UpSetR v1.4.0 (Conway et al., 2017), xlsx

v0.6.5 (Dragulescu & Arendt, 2020), writexl v1.5.0 (Ooms, 2024), and openxlsx v4.2.5 (Schauberger & Walker, 2021).

Supplementary Tables S1 to S6

Table S1. Overview of ARMS-MBON project web pages, GitHub repositories, taxonomic occurrences of data release 001 published in OBIS, and their IMIS metadata records.

**a) ARMS-MBON main webpages and GitHub documentation and data repositories**
ARMS-MBON data landing page<br>https://data.arms-mbon.org
ARMS-MBON main GitHub page<br>https://github.com/arms-mbon
Documentation repository<br>https://github.com/arms-mbon/documentation
ARMS-MBON Handbook version applied for first sampling campaign<br>https://github.com/arms-mbon/documentation/tree/main/armsmbon_handbook/old
Molecular Standard Operating Procedures (MSOP)<br>https://github.com/arms-mbon/documentation/tree/main/standard_operating_procedures
All ARMS-MBON harvested metadata and analysis data organised in folders<br>https://github.com/arms-mbon/data_workspace
All ARMS-MBON quality-controlled observatory, sampling event, image and genetic metadata<br>https://github.com/arms-mbon/data_workspace/tree/main/qualitycontrolled_data/combined
**b) *processing_batch1* repository**
Main page for all results files of PEMA bioinformatics processing of genetic data<br>https://github.com/arms-mbon/data_workspace/tree/main/analysis_data/from_pema/processing_batch1
Parameter files used for PEMA runs<br>https://github.com/arms-mbon/data_workspace/tree/main/analysis_data/from_pema/processing_batch1/parameter_files
Fasta files with ASVs/OTUs resulting from PEMA processing<br>https://github.com/arms-mbon/data_workspace/tree/main/analysis_data/from_pema/processing_batch1/fasta

ASV/OTU tables and taxonomic assignments resulting from PEMA processing https://github.com/arms-mbon/data_workspace/tree/main/analysis_data/from_pema/processing_batch1/taxonomic_assignments
Raw ASV/OTU tables resulting from PEMA processing, including blank samples and samples omitted because they did not produce ASVs/OTUs https://github.com/arms-mbon/data_workspace/tree/main/analysis_data/from_pema/processing_batch1/taxonomic_assignments_with_blanks
**c) *data_release_001* repository**
data_release_001 main page https://github.com/arms-mbon/data_release_001/tree/main
Info on observatories for which data was analysed for this data release https://github.com/arms-mbon/data_release_001/blob/main/ObservatoryData_release001.csv
Info on sampling events and material samples https://github.com/arms-mbon/data_release_001/blob/main/SamplingeventData_release001.csv
Download links for ARMS image data https://github.com/arms-mbon/data_release_001/blob/main/ImageData_release001.csv
Info on amplicon sequencing data and corresponding ENA accession numbers https://github.com/arms-mbon/data_release_001/blob/main/OmicsData_release001.csv
**d) *analysis_release_001* repository**
analysis_release_001 main page https://github.com/arms-mbon/analysis_release_001/tree/main
Parameter files used for PEMA runs https://github.com/arms-mbon/analysis_release_001/tree/main/parameter_files
Fasta files with ASVs/OTUs resulting from PEMA processing https://github.com/arms-mbon/analysis_release_001/tree/main/fasta
ASV/OTU tables and taxonomic assignments resulting from PEMA processing https://github.com/arms-mbon/analysis_release_001/tree/main/taxonomic_assignments
**d) *code_release_001* repository**
Code used for exploratory data analysis https://github.com/arms-mbon/code_release_001

**e)** ***taxonomic occurrence data sets published in OBIS***
COI<br>https://obis.org/dataset/066f002f-58d5-4687-bdb8-b39cdaef0c2b<br>*18S*<br>https://obis.org/dataset/0ada9b0c-14f5-4247-881e-9f6f62b2c165<br>*ITS*<br>https://obis.org/dataset/ddab58b2-0072-41b8-afc5-ac10d937247f
**f)** ***IMIS metadata records for taxonomic occurrences published in OBIS***
COI<br>https://doi.org/10.14284/620<br>*18S*<br>https://doi.org/10.14284/705<br>*ITS*<br>https://doi.org/10.14284/706

Table S2. Sampling effort and diversity measures per observatory for the COI and 18S marker genes. The number of samples analysed represents the number of samples remaining in the data set for each observatory after curation and filtering for data analysis. The number of reads equals the cumulative number of sequence reads in all samples used for data analysis. Number of species identified for COI was subject to the classification confidence threshold of 0.8 applied here.

Observatory	No. of ARMS units deployed	COI				18S			
		No. of samples analysed	No. of reads	ASV richness	No. of species identified	No. of samples analysed	No. of reads	OTU richness	No. of species identified
GulfOfPiran	1	3	62946	3681	67	3	63188	567	13
BelgiumCoast	2	5	14811	2318	75	5	142944	265	18
Bodo	2	6	107908	2275	168	6	177301	923	20
Gdynia	2	6	52059	2058	67	6	108049	992	25
TZS	2	6	84961	5472	70	5	143828	636	18
Getxo	3	9	7497	795	63	9	13443	253	2
Laeso	3	8	4610	365	84	9	234634	1021	25
Eilat	4	12	17421	2197	60	12	17967	753	13
Svalbard	4	11	13996	2633	94	11	410560	1309	35
Vigo	4	12	79706	2618	113	10	184248	620	18
Crete	5	13	87425	2011	79	15	325088	943	33
Limfjord	5	15	175119	3179	130	15	151523	934	19
Plymouth	5	14	107742	4317	160	15	124524	1216	29
Koster	6	18	264774	5297	246	17	425795	2136	53
Roscoff	8	24	142485	4752	215	24	352153	1756	37

Table S3. Results of Spearman's correlation analyses for the association between parameters of sampling effort (i.e., deployment duration in days and sequencing depth measured as read number for sample-wise computations; number of deployed ARMS units and number of samples included in the analysis for observatory-wise computations) and diversity measures (i.e., ASV/OTU richness and number of species identified).

Relationship investigated for Pearson's correlation	COI	18S
**Per sample**		
ASV/OTU richness vs. sequencing depth (i.e., read number)	S = 283590<br>p < .001<br>Spearman's $\rho(160)$ = .60<br>n = 162	S = 114054<br>p < .001<br>Spearman's $\rho(160)$ = .84<br>n = 162
Number of identified species vs. sequencing depth (i.e., read number)	S = 159033<br>p < .001<br>Spearman's $\rho(160)$ = .78<br>n = 162	S = 132478<br>p < .001<br>Spearman's $\rho(160)$ = .81<br>n = 162
ASV/OTU richness vs. deployment duration in days	S = 811528<br>p = .065<br>Spearman's $\rho(160)$ = -.15<br>n = 162	S = 654335<br>p = .333<br>Spearman's $\rho(160)$ = .08<br>n = 162
Number of identified species vs. deployment duration in days	S = 658469<br>p = .371<br>Spearman's $\rho(160)$ = .07<br>n = 162	S = 613801<br>p = .090<br>Spearman's $\rho(160)$ = .13<br>n = 162
**Per observatory**		
ASV/OTU richness vs. number of ARMS units deployed	S = 414.51<br>p = .350<br>Spearman's $\rho(13)$ = .26<br>n = 15	S = 193.73<br>p < .008<br>Spearman's $\rho(13)$ = .65<br>n = 15
Number of identified species vs. number of ARMS units deployed	S = 224.46<br>p = .018<br>Spearman's $\rho(13)$ = .60<br>n = 15	S = 209.14<br>p = .012<br>Spearman's $\rho(13)$ = .63<br>n = 15
ASV/OTU richness vs. number of samples remaining in the data set for ecological analysis	S = 402.29<br>p = .309<br>Spearman's $\rho(13)$ = .28<br>n = 15	S = 173.58<br>p = .004<br>Spearman's $\rho(13)$ = .69<br>n = 15
Number of identified species vs. number of samples remaining in the data set for ecological analysis	S = 230.23<br>p = .021<br>Spearman's $\rho(13)$ = .59<br>n = 15	S = 202.85<br>p = .011<br>Spearman's $\rho(13)$ = .64<br>n = 15

Table S4. Results of linear regression and generalized additive mixed modelling (GAMM) for the relationship between parameters of sampling effort (i.e., deployment duration in days and sequencing depth measured as read number for sample-wise computations; number of deployed ARMS units and number of samples included in the analysis for observatory-wise computations) and diversity measures (i.e., ASV/OTU richness and number of species identified). Linear regression was performed for associations where Spearman's correlation was significant (i.e., $p < .05$) and moderate to strong (i.e., Spearman's $\rho > .4$), see Table S3. GAMM was performed for associations including deployment duration, with the fixed effect *Deployment_Days* as smoother and the factor *Observatory* (15 levels) as random effect. Significance codes: 0 - ***; 0.001 - **; 0.01 - *.

<table>
<tr><th colspan="2">Per sample</th></tr>
<tr><th>Marker gene</th><th>ASV/OTU richness vs. sequencing depth (i.e., read number)</th></tr>
<tr><td>COI</td><td>Residuals:
Min 1Q Median 3Q Max
-651.38 -188.66 -75.57 71.04 1566.87

Coefficients:
Estimate Std. Error t value Pr(>|t|)
(Intercept) 2.209e+02 2.809e+01 7.865 5.16e-13 ***
reads 1.377e-02 1 .997e-03 6.898 1.16e-10 ***

Residual standard error: 301.6 on 160 degrees of freedom
Multiple R-squared: 0.2292, Adjusted R-squared: 0.2244
F-statistic: 47.58 on 1 and 160 DF, p-value: 1.163e-10</td></tr>
<tr><td>18S</td><td>Residuals:
Min 1Q Median 3Q Max
-368.34 -76.32 -19.77 43.07 644.21

Coefficients:
Estimate Std. Error t value Pr(>|t|)
(Intercept) 1.019e+02 1.082e+01 9.418 < 2e-16 ***
reads 2.261e-03 2.817e-04 8.026 2.04e-13 ***

Residual standard error: 122.2 on 160 degrees of freedom
Multiple R-squared: 0.287, Adjusted R-squared: 0.2826
F-statistic: 64.41 on 1 and 160 DF, p-value: 2.042e-13</td></tr>
<tr><th>Marker gene</th><th>Number of identified species vs. sequencing depth (i.e., read number)</th></tr>
<tr><td>COI</td><td>Residuals:
Min 1Q Median 3Q Max
-40.077 -12.729 -2.018 8.236 51.231

Coefficients:
Estimate Std. Error t value Pr(>|t|)
(Intercept) 1.858e+01 1.513e+00 12.282 < 2e-16 ***
reads 7.361e-04 1.075e-04 6.844 1.55e-10 ***

Residual standard error: 16.25 on 160 degrees of freedom
Multiple R-squared: 0.2265, Adjusted R-squared: 0.2216
F-statistic: 46.85 on 1 and 160 DF, p-value: 1.552e-10</td></tr>
</table>

<table>
<tr><td>18S</td><td>Residuals:
Min 1Q Median 3Q Max
-16.1862 -2.3383 -0.8963 1.2926 27.0470

Coefficients:
Estimate Std. Error t value Pr(>|t|)
(Intercept) 2.666e+00 3.768e-01 7.076 4.4e-11 ***
reads 1.044e-04 9.805e-06 10.646 < 2e-16 ***

Residual standard error: 4.253 on 160 degrees of freedom
Multiple R-squared: 0.4146, Adjusted R-squared: 0.411
F-statistic: 113.3 on 1 and 160 DF, p-value: < 2.2e-16</td></tr>
<tr><th>Marker gene</th><th>ASV/OTU richness vs. deployment duration in days</th></tr>
<tr><td>COI</td><td>GAM component:

Family: gaussian
Link function: identity

Formula:
richness ~ s(Deployment_Days)

Parametric coefficients:
Estimate Std. Error t value Pr(>|t|)
(Intercept) 376.34 49.52 7.6 2.41e-12 ***

Approximate significance of smooth terms:
edf Ref.df F p-value
s(Deployment_Days) 2.059 2.059 11.18 1.92e-05 ***

R-sq.(adj) = 0.0445
lmer.REML = 2283.4 Scale est. = 70831 n = 162

LMM component:

Linear mixed model fit by REML ['lmerMod']

REML criterion at convergence: 2283.4

Scaled residuals:
Min 1Q Median 3Q Max
-2.1438 -0.5288 -0.1362 0.3054 4.0221

Random effects:
Groups Name Variance Std.Dev. Corr
Observatory (Intercept) 1.737e+05 416.820
Deployment_Days 9.722e-01 0.986 -0.96
Xr s(Deployment_Days) 6.965e+04 263.914
Residual 7.083e+04 266.141
Number of obs: 162, groups: Observatory, 15; Xr, 8</td></tr>
</table>

<table>
<tr><td></td><td>

Fixed effects:

	Estimate	*Std. Error*	*t value*
X(Intercept)	*376.34*	*60.84*	*6.186*
Xs(Deployment_Days)Fx1	*-164.59*	*112.49*	*-1.463*

Correlation of Fixed Effects:

	X(Int)
Xs(Dpl_D)F1	*-0.198*

</td></tr>
<tr><td>18S</td><td>

GAM component:

Family: gaussian
Link function: identity

Formula:
richness ~ s(Deployment_Days)

Parametric coefficients:

	Estimate	Std. Error	t value	Pr(>|t|)
(Intercept)	*165.07*	*20.94*	*7.883*	*4.83e-13 ****

Approximate significance of smooth terms:

	edf	*Ref.df*	*F*	*p-value*
s(Deployment_Days)	*2.475*	*2.475*	*0.829*	*0.452*

R-sq.(adj) = -0.0194
lmer.REML = 2039.9 Scale est. = 15175 n = 162

LMM component:

Linear mixed model fit by REML ['lmerMod']

REML criterion at convergence: 2039.9

Scaled residuals:

Min	*1Q*	*Median*	*3Q*	*Max*
-1.5765	*-0.5983*	*-0.1643*	*0.2830*	*4.0572*

Random effects:

Groups	*Name*	*Variance*	*Std.Dev.*	*Corr*
Observatory	*(Intercept)*	*4.803e+04*	*219.1619*	
	Deployment_Days	*4.101e-01*	*0.6404*	*-0.98*
Xr	*s(Deployment_Days)*	*3.322e+04*	*182.2728*	
Residual		*1.517e+04*	*123.1861*	

Number of obs: 162, groups: Observatory, 15; Xr, 8

Fixed effects:

	Estimate	*Std. Error*	*t value*
X(Intercept)	*165.07*	*26.12*	*6.320*
Xs(Deployment_Days)Fx1	*18.79*	*73.10*	*0.257*

</td></tr>
</table>

<table>
<tr><td></td><td>Correlation of Fixed Effects:
X(Int)
Xs(Dpl_D)F1 -0.090</td></tr>
<tr><th>Marker gene</th><th>Number of identified species vs. deployment duration in days</th></tr>
<tr><td>COI</td><td>GAM component:

Family: gaussian
Link function: identity

Formula:
species ~ s(Deployment_Days)

Parametric coefficients:
Estimate Std. Error t value Pr(>|t|)
(Intercept) 24.021 3.176 7.564 2.95e-12 ***

Approximate significance of smooth terms:
edf Ref.df F p-value
s(Deployment_Days) 1.945 1.945 0.227 0.81

R-sq.(adj) = -0.00107
lmer.REML = 1316.4 Scale est. = 161.13 n = 162

LMM component:

Linear mixed model fit by REML ['lmerMod']

REML criterion at convergence: 1316.4

Scaled residuals:
Min 1Q Median 3Q Max
-2.3234 -0.6694 -0.0388 0.4748 3.8245

Random effects:
Groups Name Variance Std.Dev. Corr
Observatory (Intercept) 1.936e+02 13.91318
Deployment_Days 3.148e-03 0.05611 -0.71
Xr s(Deployment_Days) 1.396e+02 11.81663
Residual 1.611e+02 12.69350
Number of obs: 162, groups: Observatory, 15; Xr, 8

Fixed effects:
Estimate Std. Error t value
X(Intercept) 24.02056 3.55523 6.756
Xs(Deployment_Days)Fx1 -0.03411 5.65620 -0.006

Correlation of Fixed Effects:
X(Int)
Xs(Dpl_D)F1 0.290</td></tr>
</table>

18S	*GAM component:*<br><br>*Family: gaussian*<br>*Link function: identity*<br><br>*Formula:*<br>*species ~ s(Deployment_Days)*<br><br>*Parametric coefficients:*<br>*Estimate Std. Error t value Pr(>|t|)*<br>*(Intercept) 5.4938 0.7836 7.011 6.4e-11 ****<br>*---*<br>*Approximate significance of smooth terms:*<br>*edf Ref.df F p-value*<br>*s(Deployment_Days) 2.01 2.01 0.003 0.998*<br><br>*R-sq.(adj) = -0.00787*<br>*lmer.REML = 999.86 Scale est. = 23.302 n = 162*<br><br>*LMM component:*<br><br>*Linear mixed model fit by REML ['lmerMod']*<br><br>*REML criterion at convergence: 999.9*<br><br>*Scaled residuals:*<br>*Min 1Q Median 3Q Max*<br>*-1.3709 -0.6175 -0.1944 0.2434 3.9409*<br><br>*Random effects:*<br>*Groups Name Variance Std.Dev. Corr*<br>*Observatory (Intercept) 6.330e+01 7.95633*<br>*Deployment_Days 4.741e-04 0.02177 -0.98*<br>*Xr s(Deployment_Days) 2.085e+01 4.56655*<br>*Residual 2.330e+01 4.82718*<br>*Number of obs: 162, groups: Observatory, 15; Xr, 8*<br><br>*Fixed effects:*<br>*Estimate Std. Error t value*<br>*X(Intercept) 5.4938 0.9826 5.591*<br>*Xs(Deployment_Days)Fx1 0.6055 2.0781 0.291*<br><br>*Correlation of Fixed Effects:*<br>*X(Int)*<br>*Xs(Dpl_D)F1 -0.198*

<table>
<tr><th colspan="2">Per observatory</th></tr>
<tr><th>Marker gene</th><th>ASV/OTU richness vs. number of ARMS units deployed</th></tr>
<tr><td>18S</td><td>Residuals:
Min 1Q Median 3Q Max
-558.04 -264.98 12.52 255.85 736.30

Coefficients:
Estimate Std. Error t value Pr(>|t|)
(Intercept) 222.38 216.11 1.029 0.32225
arms 196.22 52.11 3.766 0.00236 **

Residual standard error: 364.5 on 13 degrees of freedom
Multiple R-squared: 0.5217, Adjusted R-squared: 0.4849
F-statistic: 14.18 on 1 and 13 DF, p-value: 0.002357</td></tr>
<tr><th>Marker gene</th><th>ASV/OTU richness vs. number of samples analysed</th></tr>
<tr><td>18S</td><td>Residuals:
Min 1Q Median 3Q Max
-585.72 -283.19 -10.09 230.12 780.79

Coefficients:
Estimate Std. Error t value Pr(>|t|)
(Intercept) 257.68 209.29 1.231 0.2400
samples 64.56 17.29 3.734 0.0025 **

Residual standard error: 366.1 on 13 degrees of freedom
Multiple R-squared: 0.5175, Adjusted R-squared: 0.4804
F-statistic: 13.94 on 1 and 13 DF, p-value: 0.002502</td></tr>
<tr><th>Marker gene</th><th>Number of identified species vs. number of ARMS units deployed</th></tr>
<tr><td>COI</td><td>Residuals:
Min 1Q Median 3Q Max
-61.274 -18.660 -5.531 11.597 92.954

Coefficients:
Estimate Std. Error t value Pr(>|t|)
(Intercept) 31.561 26.348 1.198 0.25235
arms 21.743 6.353 3.422 0.00454 **

Residual standard error: 44.44 on 13 degrees of freedom
Multiple R-squared: 0.474, Adjusted R-squared: 0.4335
F-statistic: 11.71 on 1 and 13 DF, p-value: 0.004542</td></tr>
<tr><td>18S</td><td>Residuals:
Min 1Q Median 3Q Max
-18.832 -5.745 1.305 4.030 19.755</td></tr>
</table>

	Coefficients:<br>*Estimate Std. Error t value Pr(>|t|)*<br>*(Intercept) 8.420 5.874 1.433 0.1753*<br>*arms 4.138 1.416 2.921 0.0119 **<br>---<br>*Residual standard error: 9.907 on 13 degrees of freedom*<br>*Multiple R-squared: 0.3963, Adjusted R-squared: 0.3499*<br>*F-statistic: 8.535 on 1 and 13 DF, p-value: 0.01191*
**Marker gene**	**Number of identified species vs. number of samples analysed**
COI	*Residuals:*<br>*Min 1Q Median 3Q Max*<br>*-61.800 -17.355 -7.578 9.644 91.533*<br><br>*Coefficients:*<br>*Estimate Std. Error t value Pr(>|t|)*<br>*(Intercept) 31.134 24.775 1.257 0.23098*<br>*samples 7.555 2.052 3.681 0.00277 ***<br>---<br>*Residual standard error: 42.87 on 13 degrees of freedom*<br>*Multiple R-squared: 0.5104, Adjusted R-squared: 0.4728*<br>*F-statistic: 13.55 on 1 and 13 DF, p-value: 0.002766*
18S	*Residuals:*<br>*Min 1Q Median 3Q Max*<br>*-19.496 -4.533 1.773 3.553 20.967*<br><br>*Coefficients:*<br>*Estimate Std. Error t value Pr(>|t|)*<br>*(Intercept) 9.6417 5.7948 1.664 0.1200*<br>*samples 1.3171 0.4787 2.751 0.0165 **<br>---<br>*Residual standard error: 10.14 on 13 degrees of freedom*<br>*Multiple R-squared: 0.368, Adjusted R-squared: 0.3194*<br>*F-statistic: 7.57 on 1 and 13 DF, p-value: 0.01649*

Table S5. Number of identified species (with occurrences of at least two sequence reads) across observatories listed in four different databases. Data of COI and 18S marker genes were pooled for each observatory. AMBI – species registered as very sensitive to disturbance in AZTI's Marine Biotic Index; Borja *et al.*, 2000, 2019); WRiMS – species registered as alien at the location of occurrence in the World Register of Introduced Marine Species (Costello *et al.*, 2014); IUCN/HELCOM Red List – species registered as Near Threatened, Vulnerable, Endangered or Critically Endangered in the Red Lists of the International Union for the Conservation of Nature and the Baltic Marine Environment Protection Commission (Helsinki Commission). Observatories are ordered from top to bottom by increasing number of ARMS units deployed (n = 1 for GulfofPiran, n = 8 for Roscoff).

Observatory	AMBI	WRiMS	IUCN/HELCOM Red List
GulfOfPiran	15	3	0
BelgiumCoast	17	4	0
Bodo	17	5	0
Gdynia	2	6	0
TZS	4	5	0
Getxo	8	0	0
Laeso	13	0	0
Eilat	7	0	0
Svalbard	11	0	0
Vigo	16	1	0
Crete	7	4	0
Limfjord	13	8	0
Plymouth	20	3	1
Koster	37	6	2
Roscoff	23	4	2

Table S6. Mean, standard deviation and results of statistical tests for differences in ASV/OTU richness and the number of species identified among habitats of varying degrees of anthropogenic influence for the COI and 18S data. Samples with less than 5,000 sequence reads were removed prior to the analysis and all remaining samples were rarefied to an even sequencing depth of 5,000 reads.

**Statistical measure/test**	**COI**	
	**ASV richness**	**No. of species identified**
Mean ± standard deviation	Mean / SD<br>Industrial/Semi-industrial: 259.79 / 200.10<br>Low Human Influence: 216.35 / 89.98<br>Protected: 260.00 / 172.93	Mean / SD<br>Industrial/Semi-industrial: 29.00 / 12.80<br>Low Human Influence: 26.24 / 7.55<br>Protected: 38.09 / 14.96
Shapiro-Wilk test	W = 0.89085, p < 0.001<br>log(1+x)-transformed data: W = 0.98638, p = 0.7142	W = 0.97576, p = 0.2479
ANOVA	log(1+x)-transformed data:<br>ANOVA / Df / Sum Sq / Mean Sq / F value / Pr(>F)<br>Habitat / 2 / 0.234 / 0.1171 / 0.241 / 0.787<br>Residuals / 60 / 29.2 / 0.4867	ANOVA / Df / Sum Sq / Mean Sq / F value / Pr(>F)<br>Habitat / 2 / 1577 / 788.3 / 5.043 / 0.00945<br>Residuals / 60 / 9379 / 156.3<br><br>TUKEY HSD post-hoc / diff / lwr / upr / p adj<br>LHI – (semi)industrial / -2.765 / -12.289 / 6.760 / 0.766<br>Protected – (semi)industrial / 9.091 / 0.222 / 17.959 / 0.043<br>Protected – LHI / 11.856 / 2.153 / 21.558 / 0.013
**Statistical measure/test**	**18S**	
	**OTU richness**	**No. of species identified**
Mean ± standard deviation	Mean / SD<br>Industrial/Semi-industrial: 123.00 / 77.90<br>Low Human Influence: 136.05 / 52.94<br>Protected: 165.22 / 83.72	Mean / SD<br>Industrial/Semi-industrial: 5.35 / 4.50<br>Low Human Influence: 5.81 / 3.89<br>Protected: 6.65 / 3.01
Shapiro-Wilk test	W = 0.96492, p = 0.06572	W = 0.95471, p = 0.01963<br>log(1+x)-transformed data: W = 0.93212, p = 0.001658
ANOVA/ Kruskal-Wallis	ANOVA / Df / Sum Sq / Mean Sq / F value / Pr(>F)<br>Habitat / 2 / 20349 / 10174 / 1.906 / 0.157<br>Residuals / 61 / 325555 / 5337	Kruskal-Wallis chi-squared = 2.5433, df = 2, p = 0.2804

Supplementary Figures S1 to S2

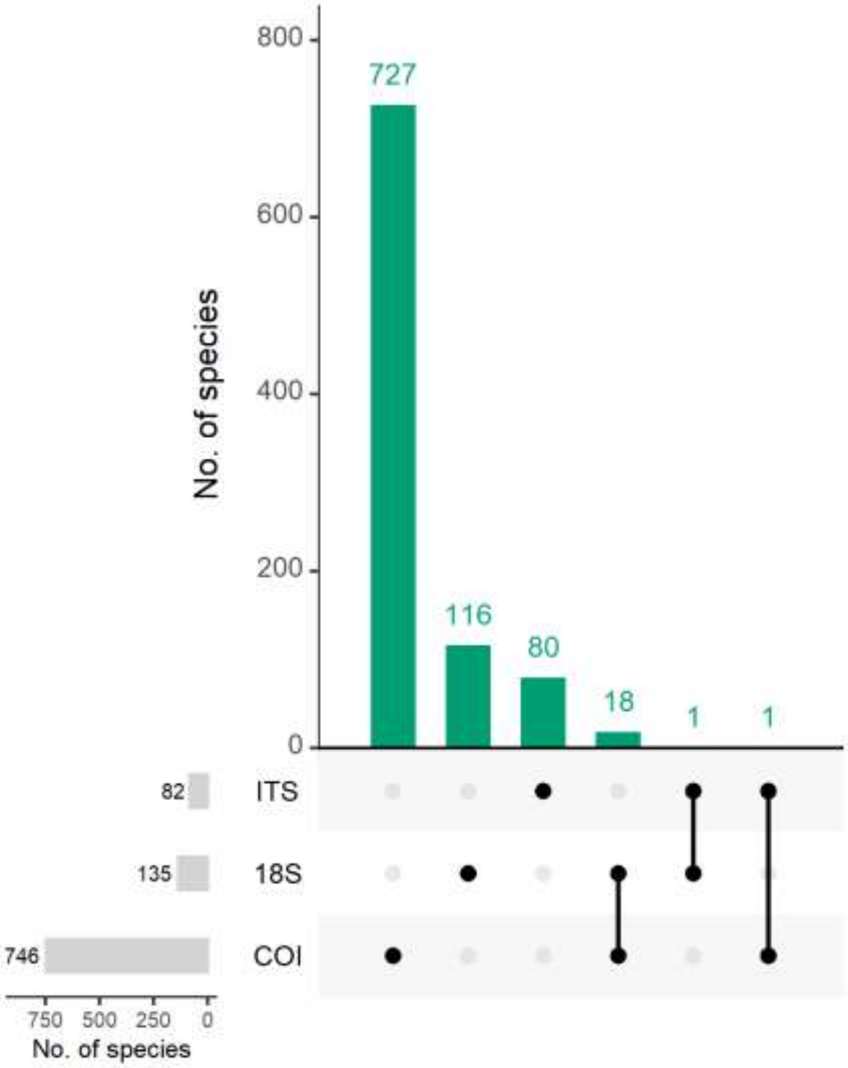

Figure S1. UpSet plot of the number of species identified using the three marker genes COI, 18S, and ITS. Green bars display the number of species identified (at the given confidence threshold applied here) that are shared across the three data sets. The matrix below the bar plot shows which combination of marker genes correspond to each bar. Bars on the left represent the total number of species identified in each marker gene's data set. No species were common to all three data sets.

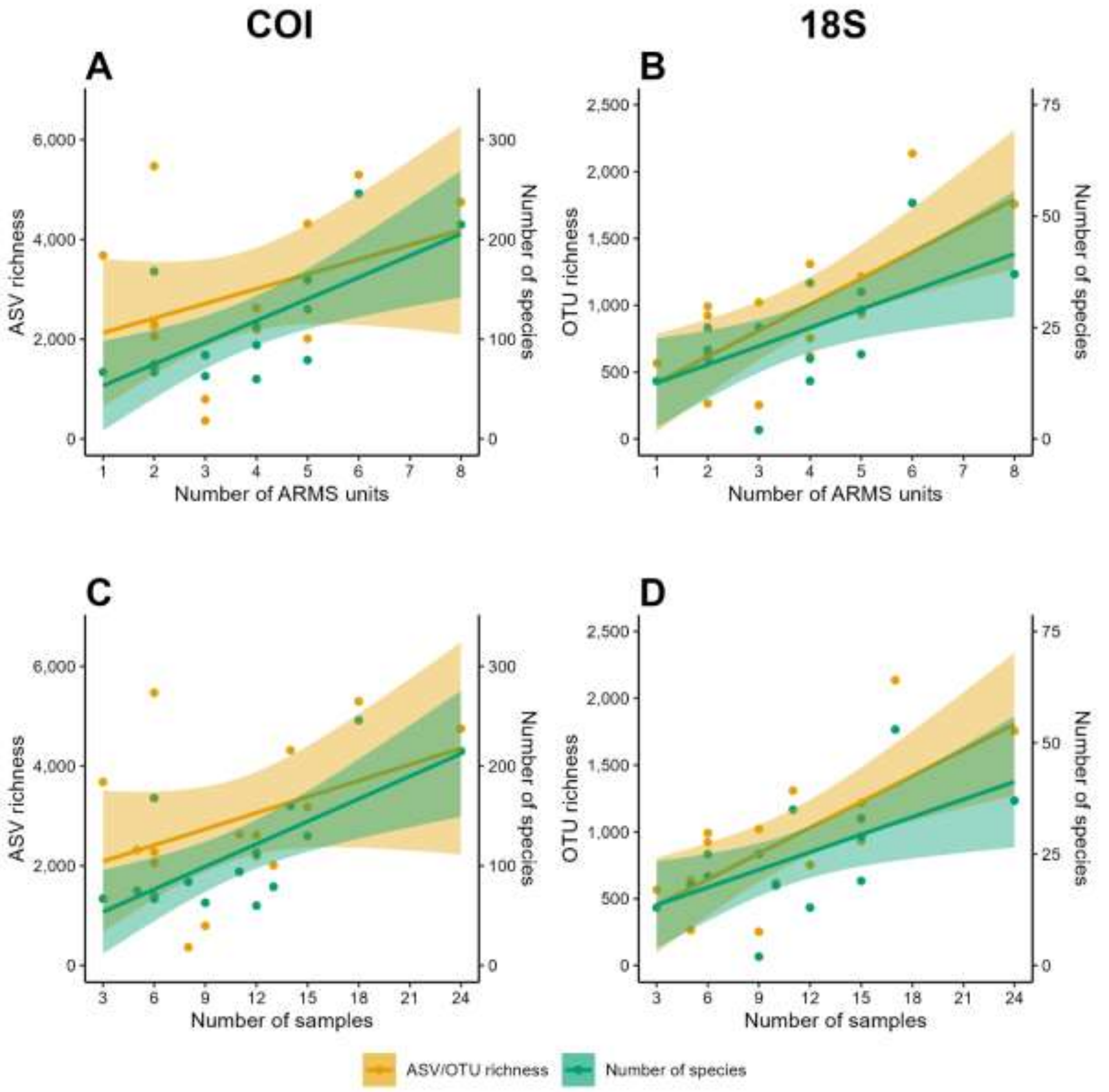

Figure S2. Relationship of ASV/OTU richness (yellow) and the number of species identified (green) for COI (**A**) and 18S (**B**) versus the number of ARMS units deployed at each observatory; and relationship of ASV/OTU richness (yellow) and the number of species identified (green) for COI (**C**) and 18S (**D**) versus the number of samples remaining in the data sets after curation for each observatory. Solid lines represent linear regression for ASV/OTU richness (yellow) and the number of species identified (green), shaded areas represent the corresponding 95% confidence intervals. Note that no significant linear correlation was found for COI ASV richness versus both sampling effort parameters in both **A** and **C**.

BIBLIOGRAPHY

Ahyong, S., Boyko, C. B., Bailly, N., Bernot, J., Bieler, R., Brandão, S. N., Daly, M., De Grave, S., Gofas, S., Hernandez, F., Hughes, L., Neubauer, T. A., Paulay, G., Boydens, B., Decock, W., Dekeyzer, S., Vandepitte, L., Vanhoorne, B., Adlard, R., … Zullini, A. (2024). World Register of Marine Species (WoRMS). WoRMS Editorial Board. https://www.marinespecies.org

Andrews, S. (2010). FastQC: A Quality Control Tool for High Throughput Sequence Data. Available online at: http://www.bioinformatics.babraham.ac.uk/projects/fastqc.

Auguie, B. (2019). egg: Extensions for “ggplot2”: Custom Geom, Custom Themes, Plot Alignment, Labelled Panels, Symmetric Scales, and Fixed Panel Size. https://cran.r-project.org/package=egg

Bankevich, A., Nurk, S., Antipov, D., Gurevich, A. A., Dvorkin, M., Kulikov, A. S., Lesin, V. M., Nikolenko, S. I., Pham, S., Prjibelski, A. D., Pyshkin, A. V., Sirotkin, A. V., Vyahhi, N., Tesler, G., Alekseyev, M. A., & Pevzner, P. A. (2012). SPAdes: A New Genome Assembly Algorithm and Its Applications to Single-Cell Sequencing. Journal of Computational Biology, 19(5), 455–477. https://doi.org/10.1089/CMB.2012.0021

Bolger, A. M., Lohse, M., & Usadel, B. (2014). Trimmomatic: a flexible trimmer for Illumina sequence data. Bioinformatics, 30(15), 2114–2120. https://doi.org/10.1093/BIOINFORMATICS/BTU170

Borja, A., Chust, G., & Muxika, I. (2019). Forever young: The successful story of a marine biotic index. Advances in Marine Biology, 82, 93–127. https://doi.org/10.1016/BS.AMB.2019.05.001

Borja, A., Franco, J., & Pérez, V. (2000). A Marine Biotic Index to Establish the Ecological Quality of Soft-Bottom Benthos Within European Estuarine and Coastal Environments. Marine Pollution Bulletin, 40(12), 1100–1114. https://doi.org/10.1016/S0025-326X(00)00061-8

Boyer, F., Mercier, C., Bonin, A., Le Bras, Y., Taberlet, P., & Coissac, E. (2016). obitools: a unix-inspired software package for DNA metabarcoding. Molecular Ecology Resources, 16(1), 176–182. https://doi.org/10.1111/1755-0998.12428

Brandt, M. I., Trouche, B., Quintric, L., Günther, B., Wincker, P., Poulain, J., & Arnaud-Haond, S. (2021). Bioinformatic pipelines combining denoising and clustering tools allow for more comprehensive prokaryotic and eukaryotic metabarcoding. Molecular Ecology Resources, 21(6), 1904–1921. https://doi.org/10.1111/1755-0998.13398

Conway, J. R., Lex, A., & Gehlenborg, N. (2017). UpSetR: an R package for the visualization of intersecting sets and their properties. Bioinformatics, 33(18), 2938–2940. https://doi.org/10.1093/bioinformatics/btx364

Costello, M. J., Ahyong, S., Bieler, R., Boudouresque, C., Desiderato, A., Downey, R., Galil, B. S., Gollasch, S., Hutchings, P., Kamburska, L., Katsanevakis, S., Kupriyanova, E., Lejeusne, C., Ma, K. C. K., Marchini, A., Occhipinti, A., Pagad, S., Panov, V. E., Poore, G. C. B., … Zhan, A. (2024). World Register of Introduced Marine Species (WRiMS). WoRMS Editorial Board. https://www.marinespecies.org/introduced

Costello, M. J., Dekeyzer, S., Galil, B., Hutchings, P., Katsanevakis, S., Pagad, S., Robinson, T., Turon, X., Vandepitte, L., Vanhoorne, B., Verfaille, K., Willan, R., & Rius, M. (2021). Introducing the World Register of Introduced Marine Species (WRiMS). Management of Biological Invasions, 12(4), 792–811. https://doi.org/10.3391/mbi.2021.12.4.02

Dragulescu, A., & Arendt, C. (2020). xlsx: Read, Write, Format Excel 2007 and Excel 97/2000/XP/2003 Files. https://cran.r-project.org/package=xlsx

Guillou, L., Bachar, D., Audic, S., Bass, D., Berney, C., Bittner, L., Boutte, C., Burgaud, G., De Vargas, C., Decelle, J., Del Campo, J., Dolan, J. R., Dunthorn, M., Edvardsen, B., Holzmann, M., Kooistra, W. H. C. F., Lara, E., Le Bescot, N., Logares, R., … Christen, R. (2013). The Protist Ribosomal Reference database (PR2): a catalog of unicellular eukaryote Small Sub-Unit rRNA sequences with curated taxonomy. Nucleic Acids Research, 41(D1), D597–D604. https://doi.org/10.1093/NAR/GKS1160

Hakimzadeh, A., Abdala Asbun, A., Albanese, D., Bernard, M., Buchner, D., Callahan, B., Caporaso, J. G., Curd, E., Djemiel, C., Brandström Durling, M., Elbrecht, V., Gold, Z., Gweon, H. S., Hajibabaei, M., Hildebrand, F., Mikryukov, V., Normandeau, E., Özkurt, E., M. Palmer, J., … Anslan, S. (2023). A pile of pipelines: An overview of the bioinformatics software for metabarcoding data analyses. Molecular Ecology Resources, 00, 1–17. https://doi.org/10.1111/1755-0998.13847

Kassambara, A. (2020). ggpubr: "ggplot2" Based Publication Ready Plots. R package version 0.4.0. https://cran.r-project.org/package=ggpubr

Lanzén, A., Jørgensen, S. L., Huson, D. H., Gorfer, M., Grindhaug, S. H., Jonassen, I., Øvreås, L., & Urich, T. (2012). CREST-Classification Resources for Environmental Sequence Tags. https://doi.org/10.1371/journal.pone.0049334

Machida, R. J., Leray, M., Ho, S. L., & Knowlton, N. (2017). Metazoan mitochondrial gene sequence reference datasets for taxonomic assignment of environmental samples. Scientific Data 2017 4:1, 4(1), 1–7. https://doi.org/10.1038/sdata.2017.27

Mahé, F., Rognes, T., Quince, C., de Vargas, C., & Dunthorn, M. (2015). Swarmv2: Highly-scalable and high-resolution amplicon clustering. PeerJ, 2015(12), e1420. https://doi.org/10.7717/PEERJ.1420

Masella, A. P., Bartram, A. K., Truszkowski, J. M., Brown, D. G., & Neufeld, J. D. (2012). PANDAseq: Paired-end assembler for illumina sequences. BMC Bioinformatics, 13(1), 1–7. https://doi.org/10.1186/1471-2105-13-31

McMurdie, P. J., & Holmes, S. (2013). phyloseq: An R Package for Reproducible Interactive Analysis and Graphics of Microbiome Census Data. PLOS ONE, 8(4), e61217. https://doi.org/10.1371/JOURNAL.PONE.0061217

Nikolenko, S. I., Korobeynikov, A. I., & Alekseyev, M. A. (2013). BayesHammer: Bayesian clustering for error correction in single-cell sequencing. BMC Genomics, 14(1), 1–11. https://doi.org/10.1186/1471-2164-14-S1-S7/TABLES/3

Nilsson, R. H., Larsson, K.-H., Taylor, A. F. S., Bengtsson-Palme, J., Jeppesen, T. S., Schigel, D., Kennedy, P., Picard, K., Gï Ockner 10, F. O., Tedersoo, L., Saar, I., & Abarenkov, K. (2018). The UNITE database for molecular identification of fungi: handling dark taxa and parallel taxonomic classifications. Nucleic Acids Research, 47, 259–264. https://doi.org/10.1093/nar/gky1022

Oksanen, J., Simpson, G. L., Blanchet, F. G., Kindt, R., Legendre, P., Minchin, P. R., O'Hara, R. B., Solymos, P., Stevens, M. H. H., Szoecs, E., Wagner, H., Barbour, M., Bedward, M., Bolker, B., Borcard, D., Carvalho, G., Chirico, M., De Caceres, M., Durand, S., … Weedon, J. (2023). vegan: Community Ecology Package. https://github.com/vegandevs/vegan

Ooms, J. (2024). writexl: Export Data Frames to Excel "xlsx" Format. https://cran.r-project.org/package=writexl

Pagès, H., Aboyoun, P., Gentleman, R., & DebRoy, S. (2020). Biostrings: Efficient manipulation of biological strings. https://bioconductor.org/packages/Biostrings

R Core Team. (2021). R: A language and environment for statistical computing. R Foundation for Statistical Computing. https://www.r-project.org/

Rognes, T., Flouri, T., Nichols, B., Quince, C., & Mahé, F. (2016). VSEARCH: A versatile open source tool for metagenomics. PeerJ, 2016(10), e2584. https://doi.org/10.7717/PEERJ.2584

RStudio Team. (2022). RStudio: Integrated Development Environment for R. RStudio, PBC. http://www.rstudio.com/

Schauberger, P., & Walker, A. (2021). openxlsx: Read, Write and Edit xlsx Files. https://cran.r-project.org/package=openxlsx

Shenoy, A. R. (2021). grafify: an R package for easy graphs, ANOVAs and post-hoc comparisons. https://doi.org/10.5281/zenodo.5136508

Wang, Q., Garrity, G. M., Tiedje, J. M., & Cole, J. R. (2007). Naïve Bayesian classifier for rapid assignment of rRNA sequences into the new bacterial taxonomy. Applied and Environmental Microbiology, 73(16), 5261–5267. https://doi.org/10.1128/AEM.00062-07

Wickham, H. (2011). The Split-Apply-Combine Strategy for Data Analysis. Journal of Statistical Software, 40(1), 1–29. https://www.jstatsoft.org/v40/i01/

Wickham, H., Averick, M., Bryan, J., Chang, W., McGowan, L., François, R., Grolemund, G., Hayes, A., Henry, L., Hester, J., Kuhn, M., Pedersen, T., Miller, E., Bache, S., Müller, K., Ooms, J., Robinson, D., Seidel, D., Spinu, V., … Yutani, H. (2019). Welcome to the Tidyverse. Journal of Open Source Software, 4(43), 1686. https://doi.org/10.21105/joss.01686

Wickham, H., Pedersen, T. L., & Seidel, D. (2023). scales: Scale Functions for Visualization. https://cran.r-project.org/package=scales

Wood, S., & Scheipl, F. (2020). gamm4: Generalized Additive Mixed Models using "mgcv" and "lme4." https://CRAN.R-project.org/package=gamm4.

Zafeiropoulos, H., Gioti, A., Ninidakis, S., Potirakis, A., Paragkamian, S., Angelova, N., Antoniou, A., Danis, T., Kaitetzidou, E., Kasapidis, P., Kristoffersen, J. B., Papadogiannis, V., Pavloudi, C., Ha, Q. V., Lagnel, J., Pattakos, N., Perantinos, G., Sidirokastritis, D., Vavilis, P., … Pafilis, E. (2021). 0s and 1s in marine molecular research: a regional HPC perspective. GigaScience, 10(8), 1–12. https://doi.org/10.1093/GIGASCIENCE/GIAB053

Zafeiropoulos, H., Viet, H. Q., Vasileiadou, K., Potirakis, A., Arvanitidis, C., Topalis, P., Pavloudi, C., & Pafilis, E. (2020). PEMA: a flexible Pipeline for Environmental DNA Metabarcoding Analysis of the 16S/18S ribosomal RNA, ITS, and COI marker genes. GigaScience, 9(3), 1–12. https://doi.org/10.1093/GIGASCIENCE/GIAA022
